# Traces of social culture in the lived experiences of emotional eating among Iranian obese women

**DOI:** 10.1186/s12889-024-19501-x

**Published:** 2024-07-24

**Authors:** Ehteram Ebrahimi, Marjan Mardani-Hamooleh, Mehdi Khezeli, Manouchehr Avatef-Fazeli, Mojtaba Habibi-Asgarabad

**Affiliations:** 1 Social Development and Health Promotion Research Center, Health Institute, Kermanshah University of Medical Sciences, Kermanshah, Iran; 2 Nursing and Midwifery Care Research Center, Department of Psychiatric Nursing, School of Nursing and Midwifery, Iran University of Medical Sciences, Tehran, Iran; 3 Department of Otorhinolaryngology, School of Medicine, Kermanshah University of Medical Sciences, Kermanshah, Iran; 4 Department of Psychology, Norwegian University of Science and Technology, Dragvoll, Trondheim 7491 Norway

**Keywords:** Emotional eating, Obesity, Social culture, Phenomenology, Iran

## Abstract

**Introduction:**

Emotional eating is a situation with harmful consequences for the physical, mental and social health of humans. In this regard, the present study aimed to explain the role of social culture in the lived experiences of emotional eating in Iranian obese women.

**Methods:**

This was a qualitative study conducted using the phenomenological approach. Purposive heterogeneous sampling method was used to select 17 women with body mass index of 30 and above who had emotional eating experience. Data were collected through semi-structured and face-to-face interviews. A guideline designed by the research team, whose content validity was confirmed by the expert panel, was used to conduct the interviews. Data were analyzed based on Diekelmann 7-step approach, and trustworthiness was evaluated by Lincoln and Guba criteria.

**Results:**

The main topic of the findings was social culture and emotional eating, which was explained by three themes: “influence of social culture”, “language culture of eating” and “the culture of eating together”. Influence of social culture had three sub-themes: “a legal and popular entertainment”, “hospitality culture: encouraging guests to overeat”, and “coping with the social stigma of thinness and obesity”. The language culture of eating had two sub-themes of “association of common infinitives” and “the symbol of swallowing anger “. Also, “culture of eating together” included 2 sub-topics with the titles “pleasant symbol of belonging and love” and “the symbol of family cohesion”.

**Conclusion:**

This study found that social culture through language, norms, and customs can initiate and/or reinforce emotional eating. The results of this study can be used in the design of interventions to improve emotional eating behavior in women by emphasizing the characteristics of Iranian social culture.

## Introduction

Today, people’s eating style is one of the most important behaviors related to health, which has led to some of the most important public health problems around the world, i.e. overweight and obesity. Emotional eating is one type of eating style, which is defined as eating in response to stress and negative emotions [1]. Evidence has shown that emotional eating is a way to manage negative emotions and cope with life’s problems. However, it is a temporary solution to cope with difficult emotional conditions and does not have a permanent role in eliminating or reducing negative emotions [2–4]. Also, emotional eating causes many harmful consequences for physical, mental and social health. For example, emotional eating is related to laryngeal reflux symptoms [5], undermined self-confidence, weakened self-efficacy in weight control, and overeating stigma [6]. Although emotional eating is observed in people with different levels of body mass index, it is more common in obese people [7], and in women compared to men [8]. Therefore, the study of emotional eating behavior in obese people and especially women has a research justification.

Research has shown that many factors cause and perpetuate emotional eating. In this regard, there is evidence that socio-cultural factors influence eating behavior [9–11]. Family and friend groups are examples of social aspects affecting eating patterns. Eating with friends and family (or acquaintances) increases people’s tendency to overeat [12], a phenomenon known as social facilitation of eating [13]. Frayn and Knäuper (2017), argue that it is difficult to determine whether emotional eating actually occurs in response to internal emotions or is more attributable to the external social context [14]. A cross-cultural study showed that culture plays a decisive role in the relationship between social and luxury food patterns and people’s positive and negative emotions [15]. Using the results of other studies, Dakanalis et al., (2023) have concluded that stigma and weight prejudice are associated with psychological problems and unhealthy eating behaviors, including emotional eating [16]. The results of a study in China showed that non-overweight people can still experience weight stigma and associated negative consequences, and that stress may explain the relationship between thinness stigma and emotional eating [17]. Peña-Fernández et al., (2015) believe that pleasant emotions are one of the socio-cultural factors that motivate eating behavior, especially during celebrations and religious ceremonies [18].

By examining the systematic review and meta-analysis studies, it can be concluded that most of the studies in this field have emphasized individual factors, especially psychological and weight components, and less has been addressed to socio-cultural issues [2, 19, 20]. Therefore, it is necessary to investigate the role of social culture components in the emergence of emotional eating behavior.

Researchers believe that understanding the underlying determinants and cultural elements that cause eating disorders can lead to better prevention measures in communities where the increase in eating disorders is alarming [21]. A review of studies shows that while emotional eating behavior is universal, it does not appear the same in different cultures [15, 21, 22]. This evidence shows that emotional eating is a behavior dependent on the cultural norms of societies. According to the definition of the American Psychological Association (APA), cultural norms are rules, values or social norms that define acceptable and appropriate behaviors within a culture. These norms are an informal understanding that determines the behavior of society members and can also be integrated in laws [23].

There is evidence that the prevalence of overweight and obesity in Iranian women is a common problem. A study in women in central Iran showed that the prevalence of BMI higher than 25 was more than 63% [24]. Another study in southern Iran showed that the total cumulative prevalence of obesity and overweight was 65.9%, and the obesity rate was higher in women than in men [25].

Considering the high prevalence of overweight and obesity in Iranian women and the possibility of its association with emotional eating, the present study was conducted with the aim of investigating the role of social culture in the lived experiences of emotional eating in Iranian obese women.

## Methods

### Design and participants

This qualitative study was conducted with the phenomenology approach in 2020. The inclusion criteria were: female gender, having emotional eating experience, and body mass index (BMI) greater than or equal to 30.00 kg/m² [26]. The participants were selected from women living in the cities of Kermanshah and Karaj. Sampling was purposeful and continued until data saturation.

### Data collection

Data were collected using in-depth semi-structured interviews individually and face-to-face. All interviews were conducted by a female researcher and audio recorded. Also, the researchers used notes at the same time as the interview to record the behavioral and emotional reactions of the participants and used these notes in the data analysis stage. Depending on the willingness of the participants, the interviews took between 30 and 60 min.

The main questions that were asked to the participants in the interview were: (1) Define one of the experiences you had about emotional eating, (2) Which of the other experiences in your life is similar to this experience you described, and what does it mean to you? (3) In what situations do you have emotional eating more and what is the relationship between these conditions and emotional eating? (4) In your opinion, what is the difference between emotional eating and normal eating? Also, exploratory questions were used to reach deeper layers of participants’ lived experiences (for example: What did you mean by…? Can you explain more? Please give an example in this regard).

### Data analysis

Data analysis began immediately after each interview. We used the seven-step method of Diekelmann et al., (1989) in analyzing the text of narratives [27] according to the following steps:

(1) The recorded conversations were transcribed verbatim in Persian immediately after each interview. Then these texts were read several times to get a general understanding for the researcher. (2) An explanatory summary was written for each of the interview texts. At this stage, meaningful units such as important and frequent words, and individual thoughts, beliefs or actions were identified and labeled in the form of different codes. (3) The researchers discussed the extracted semantic units and their contents. (4) In order to explain and eliminate any disagreements and contradictions in the interpretations, the process of going back to the texts and sometimes referring to the participants was done repeatedly (hermeneutic cycle). (5) Interpretive summaries and semantic units similar to integration and combined analysis were done. As a result, by categorizing similar semantic units, sub-themes and main themes were formed. (6) A final commentary or structural statement was written that expresses the connection between the extracted sub-themes and themes. (7) A draft of themes and sub-themes were given to the members of the research team and an external reviewer familiar with phenomenological study, and their suggestions were included in the final version of the report.

### Trustworthiness

To measure the rigor and trustworthiness of the research data, the five criteria of Lincoln and Guba (1988) were used [28], as follows:

To achieve the credibility criterion, strategies such as allocating enough time for data collection, long-term and continuous involvement of researchers with data, debriefing by peer, and member check methods were applied. In order to comply with dependability, the researchers allowed the participants to fully express their experiences and interpretations, keeping in mind the purpose of the research. This allowed the findings to be derived from the meaning of life with the phenomenon of emotional eating. Audit trail and peer review were also used to control the dependability of data. In order to achieve the confirmability criterion, all the extracted data and contents were checked by the research team members and the external reviewer in order to minimize the influence of the personal opinions of the researchers. To comply with the transferability of the data, a database including the description and classification of the study data was created to provide a basis for the judgment and evaluation of the readers. Finally, in order to achieve the criterion of authenticity, the participants’ speech was recorded and transcribed along with the emotions and behaviors recorded during the conversations. This helps to convey the voice and feelings and life of the participants as they lived in the report.

### Ethical considerations

In each interview, the researcher provided explanations about the objectives of the research and received informed consent from the participants, assuring the people that their personal information will remain confidential. The interviewees were informed that they are allowed to ask any questions at any time during the interview. This study received ethics approval from the Research Ethics Committee Kermanshah University of Medical Sciences (No.IR.KUMS.REC.1402.052).

## Results

This study was conducted with the participation of 17 obese women from the cities of Kermanshah (9 people) and Karaj (8 people) with an average age of 39 years. The body mass index of women was between 30.06 and 35.40. The lowest and highest level of education among the participants was fifth grade and master’s degree, respectively. In terms of ethnicity, they were Kurds (8 people), Persians (7 people) and Turks (2 people). Seven of the interviews were conducted in comprehensive health centers. The rest of the participants entered the study through the introduction of colleagues, fellow academics and relatives, as well as the invitation of the WhatsApp social network group, and the interviews were conducted at the participants’ homes.

In the analysis of the interviews, a structural pattern emerged entitled: “traces of social culture in the lived experiences of emotional eating in Iranian obese women”. This structural model was obtained by combining three themes including “influence of social culture”, “language culture of eating” and “the culture of eating together” (Table 1).

**Table 1:** Themes and sub-themes extracted from the interviews

Themes	Sub-Theme
***Influence of social culture***	• *A legal and popular entertainment* • *Hospitality culture: encouraging guests to overeat* • *Coping with the social stigma of thinness and obesity*
***Language culture of eating***	• *Association of common infinitives* • *Symbol of swallowing anger*
***The culture of eating together***	• *pleasant symbol of belonging* • *love” and “family cohesion symbol*

### Influence of social culture

The analysis of the participants’ statements showed that sometimes emotional eating is influenced by the society’s culture. This means that people do emotional eating because of negative emotions derived from cultural norms. In this regard, three sub-theme were merged with included “A legal and popular entertainment”, “hospitality culture: encouraging guests to overeat”, and “coping with the social stigma of thinness and obesity”. Finally, the theme of “influence of social culture” was formed following the combination of these sub-themes.

#### A legal and popular entertainment

according to some participants, eating is an acceptable recreation in the cultural and legal norms of society. In a situation where the variety of recreational facilities for these women was low, they used this solution to improve their mood and feel better. According to them, eating is a recreation that has no legal or cultural prohibition in society.

*I think that eating is the highest pleasure in Iran*,* because in [our] society there is no space for recreation and mental relaxation*,* especially for women. For example*,* we cannot go to a park and sing and play easily (Participant 14)*

*I think the reason is cultural*,* because we don’t have any other recreation*,* for example*,* I can’t go to the swimming pool with my husband*,* and there is no disco where we can go to dance. Anyway*,* going to a restaurant and eating has become our pastime. (Participant 17)*

***Hospitality culture: encouraging guests to overeat***: One of the participants in tracing the roots of emotional eating mentioned memories that at parties after being full, the host insisted on eating more food and she overate.

*When I was a child*,* whenever we went to a party*,* my uncle’s wife insisted that you should eat a lot (Participant 2)*

She believed that these experiences led to her use the strategy of eating to relieve emotions whenever she was under the pressure of negative emotions.

#### Coping with the social stigma of thinness and obesity

Some participants said that at some point in their lives, people have ridiculed and judged them for being too thin. One of the reasons for emotional eating in them was trying to escape from the negative emotions caused by the thinness stigma and finding a good feeling about their physical appearance. Of course, this effort has led to their obesity over time.

*When we were children*,* we were constantly told that you are fragile*,* you will break if touched*,* and that’s why I wanted to gain some weight. I wanted to gain some weight to become more beautiful and handsome. (Participant 13)*

On the contrary, some other participants had eaten more to alleviate the negative emotions related to the labels and insults of others for their obesity. Their emotional eating behavior is caused by the negative influence of others around them on their emotions.

*My husband always said I don’t like your fat body. Once at his father’s house*,* I wore a colored dress. I noticed that he was staring at me with serious face. I said to him at night: What was your problem? He said: you disgraced me with your fatty figure. He humiliated me a lot and it made me overeat again (Participant 8)*

### Language culture of eating

Analysis of the statements of some participants showed that sometimes their emotional eating is affected by the language they speak. In this regard, a theme titled “linguistic culture of eating” was obtained, which indicates the words that highlight the culture of eating in their language. This theme was formed from the combination of two sub-themes including “association of common infinitives” and “symbol of swallowing anger” (Table 1).

#### Association of the common infinitives

In many languages and dialects of the Iranian society, a number of verbs related to negative emotions are two-part verbs in which “to eat” is placed in the second part, for example “eating sadness” to describe feeling sad. This point applies to the Kurdish and Persian languages that most of the participants of this research spoke in these languages.

*Because in our language “to be sad (eating sad)” and “to eat” is the infinitive of eating*,* I think when we are sad; our mind moves us towards eating food. For example*,* whenever my siblings or I are sad*,* my mother brings snacks and tells us not to be sad (don’t eat sad)*,* children*,* eat snacks to calm your nerves. In our culture*,* eating is exactly equal to grieving*,* and instead of grieving*,* we can eat. (Participant 14)*

According to this woman, because in her language the verb “eating sad” is used instead of the verb “to be sad”, therefore being sad is associated with eating food, and this makes emotional eating easy for her.

#### Symbol of swallowing anger

In most Iranian languages and dialects, the expressions “eating anger” and “swallowing anger” are used for the concept of “reducing and eliminating anger”. In this regard, a woman likened her binge eating to opening her stomach to reduce her anger.

*When I get angry*,* unconsciously*,* I eat more. I don’t realize it at all*,* but I’m eating a lot. It’s like my stomach opens up so that the anger in my brain decreases (Participant 9)*

### The culture of eating together

According to the participants, one action that was usually done among their families, friends and relatives to improve people’s condition is to use the method of “eating together”. Based on this, the theme of “eating together culture” emerged. This theme was formed by two sub-themes including “pleasant symbol of belonging and love” and “family cohesion symbol” (Table 1).

#### Pleasant symbol of belonging and love

According to some participants, eating together was a pleasant solution that worked to improve people’s mood by evoking the feeling of belonging and love. Based on this, the sub-theme of “pleasant symbol of belonging and love” was formed.

One of the participants expressed her memories about eating together as evoking the joy of being together and the feeling of love and belonging among her family as follows:

*Eating in general gives me a very positive and good feeling*,* because I remember the time when we were all together*,* I remember my childhood when we were all together and in that group*,* no one ate with sadness*,* everyone ate happily. I feel that it [my emotional eating] originates from here because for me*,* good times were usually eating at parties (Participant 14).*

Another participant described the times when they are eating together with their relatives as the good times of life:

*A good day for us is when we are together and we are eating delicious food (Participant 17).*

#### The symbol of family cohesion

It was inferred from the statements of some participants that in their families, eating together was used to resolve concerns related to the feeling of weakening family cohesion. Based on this, the sub-theme “symbol of family cohesion” was formed.

One participant used eating with her husband as a means of getting closer to him and coping with feelings of insecurity in her marital relationship:

*I used to sit next to him and eat with him. I was saying to myself*,* well*,* we are together a little bit. Maybe I saw this closeness in eating. I didn’t understand his point of view*,* I felt like I was going to a dead end*,* this was the only way left to improve my mood. I felt the intimacy of those moments and I tried not to lose it (Participant 1).*

Another participant said that in their family, eating together was used to hide family conflicts and relieve the pain caused by these conflicts:

*Whenever my mother wanted to reconcile*,* she would cook a good meal at home. She thought that a woman’s art is to prepare food and gather her family members together (participant 8).*

## Discussion

The concept of social culture is a complex set of meanings, habits, values, and behaviors that are accepted by one or more social groups [29]. In this qualitative study, we investigated the role of society’s culture in the emotional eating behavior of Iranian obese women by analyzing the interviews. Based on the findings, we identified a structural pattern called traces of social culture in the emotional eating experiences of Iranian obese women. We obtained this structural model by linking the three themes including “influence of social culture”, “language culture of eating “and “culture of eating together “.

The results of this study showed that the society’s culture has influenced the emotional eating of the participants. We found that social judgments affect the cultural norms of the society, and the negative judgments of others or relatives make the norms more important. Therefore, obese women turned to emotional eating to relieve or prevent negative emotions arising from facing social judgments. Similarly, the findings of a qualitative study in the United States showed that social influence is effective on the occurrence of emotional eating behavior through rejection and social judgment [30]. Also, the results of a review study showed that social judgments strengthen the social norms of eating [31].

The sub-theme of “a legal and popular entertainment” refers to a type of entertainment that is both accepted by society and not considered a crime. According to the findings of this study, in the culture of Iran, eating is considered as a legal and popular entertainment. Of course, considering the legal restrictions in Iran, the term legal and popular entertainment has its own meaning in the form of cultural norms and laws of the country of Iran. We did not find a study that reported a similar result. However, the important issue is that eating as an acceptable entertainment in popular culture (especially for women) and a way to improve people’s mood can accelerate the process of weight gain in societies including Iran [32]. In this regard, evidence shows that public policies can be considered a powerful tool to create supportive environments for healthy eating [33]. Also, policymakers can provide and promote more diverse and healthier recreational facilities, so that people in the society, especially women, can use these recreational facilities to improve their mood.

One of the important results of this study is the problem of insisting on overeating guests as a hospitality culture in Iran. According to similar studies, this problem can be confirmed in other societies and cultures. A qualitative study in the Netherlands analyzed social and cultural influences on food intake in 2 non-Western immigrant groups in the city of Amsterdam. The participants were Turkish and Moroccan immigrant young adults. In the findings of that study, one of the themes was hospitality, which was rooted in the cultural and religious tradition of both groups. In both Turkish and Moroccan cultures, it is common for the host to insist that the guests eat a lot. Refusal to eat may be considered an insult to the host. Therefore, since the social norms of these cultures make it difficult for guests to manage eating, the traditional culture can contribute to overeating and ultimately overweight [34]. According to the findings of another qualitative study in Fiji, in the cultural norms of the natives of this island, hosts usually encouraged guests to overeat. Guests also forced themselves to overeat even when they felt full, to avoid disrespect, or fear of not being appreciated for the time, effort, and money the hosts had spent preparing the meal [11]. According to one of the participants of the present study, in this situation, overeating reduces the psychological pressure caused by the insistence of the host and brings a feeling of gratitude to the host. This situation leads to emotional eating because it causes her to get used to overeating to reduce his psychological pressure.

Another important sub-theme of this study was “coping with the social stigma of thinness and obesity”. In Western culture, the perceived socio-cultural pressure on thinness has been mainly studied. Strenger et al., (2016) believe that women experience an increase in emotional eating and consequently eating disorder symptoms following social and cultural pressure to be thin [35]. Romano et al., (2018) also showed that there is a relationship between perceived overweight and the tendency to overeat with concerns about weight stigma, where participants with perceived overweight have more concerns [36]. Conversely, according to the results of a qualitative study of indigenous Fijian women, larger body size of family members indicates a successful mother or wife, and women whose family members are thin are considered irresponsible for feeding the family [11]. Also, the results of a qualitative research on Pakistani students showed that the belief “body not too fat and not too thin” has more influence in the society [37]. This is consistent with the results of the current study that both thinness and obesity are considered stigmatizing. In this regard, Kiang and Harter (2006) have previously shown that a high emphasis on sociocultural values ​​related to appearance leads to less satisfaction with appearance, which in turn leads to eating disorder behaviors [38].

The term “linguistic culture” used in the current research to name the theme related to the influence of language in the occurrence of emotional eating behavior is one of the specialized terms of the field of linguistic sociology and anthropology of language. Schiffman defines linguistic culture as: the set of ideas, values, beliefs, attitudes, prejudices, legends and religious rules that the users of a language are influenced by when using their language [39]. It seems that the influence of linguistic culture on emotional eating has not been investigated in the research background of emotional eating behavior. For this reason, no research evidence was found consistent or inconsistent with this theme and its sub-themes, i.e. “association of the common infinitive” and “the symbol of swallowing anger”. However, according to evidence, language can influence people’s behavioral performance, including health behaviors such as eating and weight management [40]. Also, a study in the United States found that Latino and Asian adolescents with low or healthy weight, whose primary language is not English, are at increased risk of distorting weight perception. Distorted weight perception, especially overestimation of weight status, is common in adolescents and may lead to eating disorders. According to the results of the mentioned study, less acculturation was associated with a greater likelihood of distorted weight perception in Latino and Asian adolescents compared to whites [41]. Based on this evidence, it can be assumed that language probably affects people’s eating behavior, which includes emotional eating behavior. In order to investigate this hypothesis, it is necessary to conduct more researches, especially quantitative research.

The participants in the present study stated that eating together is effective in relieving their negative emotions. The results of a review study similarly showed that eating alone is one of several risk factors for malnutrition in the elderly and confirms the importance of eating together in the lives of the elderly [42]. In the current study, the importance of eating together was revealed in two sub-themes. One was the theme “pleasant symbol of belonging and love “. In a phenomenological study in the United States, participants’ interpretations showed that food can be considered a way to solve problems related to the human need to belong and experience closer relationships [43]. In another phenomenological study in England by Close (2013), several participants introduced eating at family parties as strengthening the relationship with food to provide comfort and happiness. According to them, in adulthood, the need for support and love can trigger emotional eating through fun in happier situations [44]. As mentioned in the results section, the second sub-theme was “the symbol of family cohesion”. A review compared qualitative studies that analyzed the determinants of food choice using a cultural lens in mainland China and Chinese immigrants living in Western countries. One of the themes identified in the findings of that review was “desire to maintain harmony in families/communities.” Participants reported eating junk food or overeating to avoid conflict and maintain harmony in families/communities [45]. Also, the results of a qualitative study of Chinese diabetics showed that almost half of the participants neglected their physical needs to avoid family problems/conflicts that resulted in overeating or eating the unhealthy foods [46]. Similarly, a study in Taiwan with the elderly found that 40% of participants chose to follow family dietary rules and sacrificed themselves for the family [47].

In this study, due to the qualitative approach, small sample size, and specific Iranian setting, the generalization of the results should be done with caution. The outbreak of COVID-19 at the time of the study caused limitations in data collection. It is possible that the fear of infection with COVID − 19 has reduced the participation of certain groups of women with emotional eating behavior. In this regard, while explaining the conditions and objectives of the study, the interviewer followed the health protocols during the interviews. However, this study provided a good insight into the role of social culture in emotional eating of Iranian women. In this study, there were participants from three Iranian subcultures including Kurds, Turks, and Persians, which can be mentioned as a strength and factor in the richness of the findings.

## Conclusions

This study highlighted the role of culture-dependent social norms in the development of emotional eating behavior and showed that social cultural norms can initiate and/or strengthen emotional eating behavior with the aim of relieving stress and negative emotions. This study also warns that social cultural factors that may create obstacles in the treatment process should be considered in the interventions that are carried out to reduce the weight of obese people, especially women, through the management of emotional eating behavior. The results of this study can be used in the design of interventions to improve emotional eating behavior in women by emphasizing the characteristics of Iranian social culture.

## Data Availability

The data sets used and analyzed in this study are available from the corresponding author on reasonable request.

## Abbreviations

APA: American Psychological Association
BMI: Body mass index
